# Do individuals’ attitudes toward immigrants affect their own health? Analysis of 15 European countries

**DOI:** 10.1093/eurpub/ckab212

**Published:** 2021-12-30

**Authors:** Sara Pinillos-Franco, Ichiro Kawachi

**Affiliations:** 1 Department of Economic Analysis, Universidad Autónoma de Madrid, Madrid, Spain; 2 Department of Health and Social Behavior, Harvard School of Public Health, Boston, MA, USA

## Abstract

**Background:**

Hostility toward others is related to negative emotions, which is hypothesized to have negative health consequences. In this article, we sought to test the relationship between individuals’ attitudes toward immigrants and their self-rated health (SRH) across time in large dataset of 15 European countries.

**Methods:**

We used the 2010, 2012, 2014 and 2016 waves of the European Social Survey, which include information about attitudes toward immigrants and individuals’ SRH.

**Results:**

Combining all countries and survey years, we found an association between negative attitudes toward immigrants and fair/poor SRH. However, when analyzing the relationship within each country, we found variations (depending on the year of the survey) in some countries, such as Hungary, Poland, Portugal, Sweden and Slovenia. Additionally, we found that there are more middle class individuals who hold unfavorable attitudes toward immigrants over time.

**Conclusions:**

In general, poor attitudes toward immigrants are associated with higher odds of reporting fair/poor health, although the ‘health advantage’ of those with favorable attitudes is diminishing over time. However, in some countries, this relationship is contingent on when the survey was conducted, possibly reflecting the changing composition of individuals who hold unfavorable attitudes toward immigrants.

## Introduction

The EU region witnessed a marked increase in anti-immigrant sentiment in the wake of the Great Recession of 2007–10. Competitive threat theory suggests that anti-immigrant attitudes increase when adverse economic circumstances intensify competition with immigrants for jobs,^1^ placement in schools, social welfare benefits, health care spending^2^ and other scarce resources.^3^ Far-right political parties have capitalized on this anxiety by stoking prejudice and hate against immigrants, refugees, asylum seekers and other perceived ‘outsider’ groups.^4–6^ In the 2014 EU Parliament Election, far-right political parties reached the European Parliament in countries, such as France, Denmark, Italy, Poland, Greece, Hungary, Finland, Sweden, Belgium and Germany (https://www.enar-eu.org/National-and-European-elections-and-the-rise-of-far-right-parties). In other countries, such as Spain, Switzerland, Norway and Slovenia, far-right political parties also reached the Parliament of their respective countries during the same period. 

While previous studies have focused on the detrimental effects on the groups targeted by anti-immigrant prejudice (e.g. hate crimes), less is known about the health of individuals who harbor such sentiments. In an analysis of survey data from 18 European countries included in the 2016 European Social Survey (ESS), Backhaus et al.^7^ found that individuals who voted for right-wing populist parties were 40% more likely to report fair/poor health compared with traditional conservatives. It is unclear whether negative views toward immigrants is a marker for other characteristics, which correlate with poor health (e.g. job insecurity and financial hardship), or whether there is a direct effect of harboring hostile attitudes on mental and physical health.

Attitudes toward immigrants are mainly developed during adolescence and seem to be stable across time.^8^ The process of ‘othering’ can lead to either negative or positive consequences depending on whether it is exclusionary or inclusionary.^9^^,^^10^ Exclusionary othering involves the process of coming to view others as different due to their personal traits, such as skin color, physical disabilities, language and/or gender, precluding relationships and leading to exclusion, marginalization or alienation of the group seen as being ‘different’. Conversely, inclusionary othering leads to higher consciousness, sense of community, shared power and inclusion.

Negative attitudes toward immigrants may be thus a consequence of exclusionary othering, as it is a process often based on ethnic and/or racial biases.^9^^,^^10^ These attitudes are characterized by prejudice, stereotypes or ignorance toward other’s values, customs or culture, and can lead to ‘intergroup anxiety’, i.e. anxiety stemming from contacting ‘others’ from a different group.^11^^,^^12^ In an experimental study involving interactions (in a laboratory setting) between Latinos and Whites found higher levels of cortisol reactivity among individuals who harbored prior prejudices toward the other group or were worried about being rejected by the other group.^13^ Negative attitudes toward immigrants may not only lead to stress and anxiety, but also to increased frequency of experiencing negative emotions, such as fear, anger and resentment,^14^ which have been linked to physical and mental health problems,^15^ including cardiovascular disease events.^16^

In this article, we sought to test the hypothesis that individuals’ attitudes toward immigrants is detrimentally associated their self-rated health (SRH) in the general population across Europe and over time. The period selected was from 2010, as it is when far-right political parties started obtaining representation across different European countries. We focused on SRH as it is the only self-reported health measure available on the ESSs. On the other hand, the validity of the single-item assessment has been previously established based on its ability to predict subsequent hospitalization and mortality.^17^^,^^18^

Our hypothesis is that there is a positive association between favorable attitudes toward immigrants and higher SRH, ‘net’ of individual socioeconomic circumstances and independent of the country of individuals’ residence.

## Methods

We used the 2010, 2012, 2014 and 2016 waves of the ESS available online at www.europeansocialsurvey.org/. The ESS is a repeated cross-sectional, cross-national survey implemented in 2001, which is conducted through face-to-face interviews every 2 years. The ESS is representative of all individuals aged 15 and over living in private households in each country. A random probability sample is selected from the residential registries in each country.

We selected the 15 European countries, which participated in all waves, i.e. Belgium, Switzerland, Germany, Spain, Finland, France, UK, Hungary, Ireland, the Netherlands, Norway, Poland, Portugal, Sweden and Slovenia. A total of 113 744 individuals participated in the four waves of the survey living in the above mentioned countries aged between 14 and 104 years.

To conduct our analysis, we selected cross-sectional samples from 2010 due to the salience of immigration across Europe starting from 2009. As the dependent variable, we included individuals’ SRH and, as our independent variable, a scale to represent attitudes toward immigrants.

Individuals’ general health was assessed using the ‘self-rated health’ status. Participants are asked ‘How is your health in general? Would you say it is …’ and they can choose among five categories ‘Very good’, ‘Good’, ‘Fair’, ‘Bad’ or ‘Very bad’. We dichotomized this variable so that it takes a value of 1 when the individual’s health is fair/poor, i.e. when his/her health is ‘fair’, ‘bad’ or ‘very bad’; and 0 otherwise.

To measure individuals’ ‘attitudes toward immigrants’, we selected six questions from the survey that appeared in all waves of the ESS: (i) to what extent do you think (your country) should allow people of the majority race/ethnic from (another country) people to come and live here? (ii) How about people of a minority race/ethnic group from (another country) people? (iii) How about people from the poorer countries outside Europe?, (iv) Would you say it is generally bad or good for (your country’s) economy that people come to live here from other countries? (v) Would you say that (your country’s) cultural life is generally undermined or enriched by people coming to live here from other countries? (vi) Is (your country) made a worse or a better place to live by people coming to live here from other countries? Questions 1, 2 and 3 have four possible answers: ‘Allow many to come and live here’, ‘Allow some’, ‘Allow a few’ or ‘Allow none’, and Questions 4, 5 and 6 have a response range from 0 (‘worst place to live’) to 10 (‘better place to live’).

We reordered the responses to the first three questions so that they took values from less openness to more openness to immigration (to be consistent with the last three items). We then summed the responses to create a six-item scale and confirmed that the resulting scale had high internal consistency reliability (‘Cronbach alpha: 0.83’). The scale has a range from 0.5 (worst attitude toward immigrants) to 7.0 (most positive attitude toward immigrants).

Before calculating this scale, we performed a listwise deletion of missing values. The final sample comprised 104 279 individuals.

To test the relationship between fair/poor SRH and attitudes toward immigrants, we estimated a local weighted linear regression for each point of the dataset and represented it using Loess curves. In our case, each point of our independent variable (i.e. attitudes toward immigrants) uses the 80% of the dataset to fit a value. This process is made with all the points of the dataset to assign them a weight, therefore, the most weighted points would be those closest to the focal point, i.e. the point from which the process started, and the least weighted those which are further from this focal point. We have thus weights based on the distance between the points in our independent variable (*x*-axis), and weights based on the distance between the ‘old point’ and the previously calculated ‘new point’ (*y*-axis). In this case, if the ‘old point’ is close to the ‘new point’ the assigned weight will be high and, if both points are far, the weights will be low. This latter weights allow us to reduce the impact of outliers and smooth the curve, showing the relationship between fair/poor SRH and attitudes toward immigrants.

We also presented the basic descriptive statistics of whole sample and of individuals depending on their attitudes toward immigrants. To do this, we selected, on the one hand, those individuals who reported a score below the arithmetic mean of the variable ‘attitudes toward immigrants’, i.e. a score of 3.75 out of 7 or below; and from the other side, those individuals who reported a score above the arithmetic mean. This allows us to distinguish between individuals with ‘negative attitudes toward immigrants’ from individuals with ‘positive attitudes toward immigrants’.

## Results

Table 1 shows the samples sizes per country and year, and table 2 the descriptive statistics of the whole sample. More than 30% of the sample reported fair/poor health across years, although there was a declining trend over time. The average age increased from around 47–49 years in the last wave. There were more females than males in all waves and almost 90% of individuals were native in their respective countries. Attitudes toward immigrants were, in general, more positive than negative and have been improving over time. Individuals had an average level of education (upper secondary level) and were actively working (around 80%). Their income mainly came from wages and salaries (around 60% of the sample) and this allowed the majority of individuals to cope with their lives without problems.

**Table 1:** Sample size after listwise deletion of missing values

Year	2010	2012	2014	2016	Total
Country					
EU-15	25 967	27 501	25 528	25 283	104 279
Belgium	1665	1837	1733	1745	6980
Switzerland	1404	1380	1439	1403	5626
Germany	2728	2816	2915	2745	11 204
Spain	1712	1734	1592	1625	6663
Finland	1805	2113	1994	1842	7754
France	1659	1901	1816	1968	7344
UK	2198	2092	2130	1840	8260
Hungary	1273	1572	1388	1318	5551
Ireland	2404	2479	2146	2582	9611
Netherlands	1698	1729	1805	1551	6783
Norway	1509	1582	1390	1489	5970
Poland	1448	1574	1299	1357	5678
Portugal	1818	1846	1151	1175	5990
Sweden	1391	1739	1678	1431	6239
Slovenia	1255	1107	1052	1212	4626

**Table 2:** Descriptive statistics of the whole sample by year

	2010	2012	2014	2016
Fair/poor SRH	32.37%	31.24%	31.29%	31.11%
Attitudes toward immigrants (min. 0.5–max. 7)	3.92	4.03	4.04	4.14
Age	47.64	48.43	49.06	49.32
Female	51.82%	52.06%	51.36%	51.14%
Foreign-born	9.43%	9.78%	10.07%	10.64%
Educational attainment				
ISCED 0	2.55%	2.27%	1.64%	1.42%
ISCED 1	12.50%	11.65%	9.89%	8.99%
ISCED 2	19.40%	18.80%	17.99%	16.72%
ISCED 3	33.23%	33.65%	33.03%	33.25%
ISCED 4	4.76%	5.24%	5.70%	5.64%
ISCED 5	7.53%	7.86%	8.68%	8.27%
ISCED 6	9.62%	9.52%	10.67%	12.19%
ISCED 7	9.59%	10.08%	11.41%	12.29%
ISCED 8	0.81%	0.93%	0.99%	1.23%
Labor market situation				
Employee	79.29%	79.31%	79.89%	80.29%
Self-employed	10.65%	10.86%	11.09%	11.39%
Family business	1.32%	1.80%	1.69%	1.87%
Other situation	8.73%	8.03%	7.33%	6.45%
Perceptions about income				
Living comfortably on present income	32.64%	31.47%	36.33%	39.72%
Coping on present income	46.57%	45.61%	45.30%	45.38%
Difficult on present income	15.40%	17.06%	14.28%	11.65%
Very difficult on present income	5.40%	5.86%	4.08%	3.24%
Main source of household income				
Wages or salaries	59.00%	57.64%	58.11%	59.16%
Income from self-employment (excluding farming)	5.57%	5.41%	5.66%	5.65%
Income from farming	1.25%	1.22%	1.22%	1.26%
Pensions	25.41%	26.19%	26.40%	26.12%
Unemployment/redundancy benefit	3.51%	3.84%	2.66%	2.36%
Any other social benefits or grants	3.52%	3.77%	3.82%	3.51%
Income from investments, savings etc.	0.43%	0.62%	0.72%	0.69%
Income from other sources	1.32%	1.32%	1.40%	1.26%

**Figure 1:**
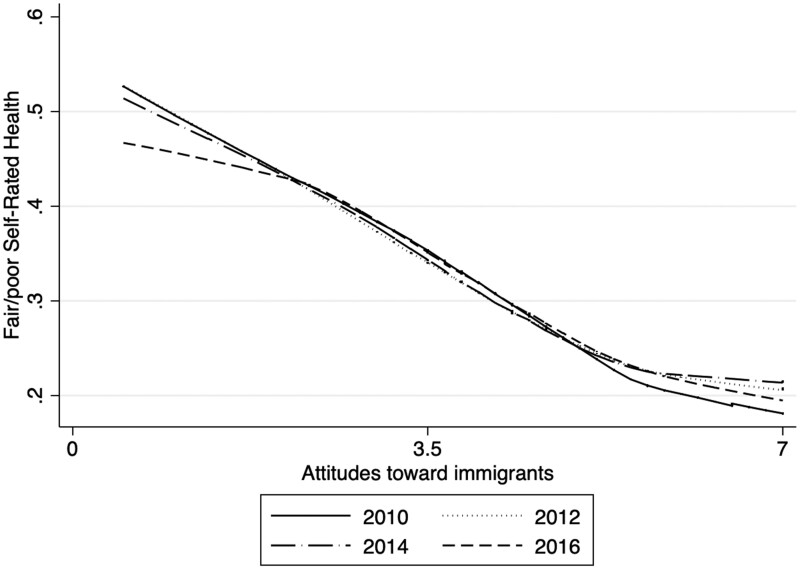
Relationship between fair/poor SRH and attitudes toward immigrants

In general, considering all countries and years together, the relationship between attitudes toward immigrants and fair/poor SRH was almost linear (figure 1). Negative attitudes toward immigrants and fair/poor SRH were strongly correlated (rs: −0.1636; Prob > |*t*| = 0.00), indicating that as attitudes toward immigrants becomes more favorable, the prevalence of reporting fair/poor SRH is lower. Two instances of departures from linearity were: (i) in 2010, when those with favorable attitudes of immigrants had significantly lower prevalence of poor SRH compared to subsequent years, and (ii) in 2016, when those with unfavorable views of immigrants reported significantly lower prevalence of poor SRH compared to previous years. As a result, the association between negative attitudes toward immigrants and poor SRH seems to have diminished in the most recent survey year, while the ‘health dividend’ from having a positive attitude toward immigrants seems to have disappeared after 2010.

**Figure 2:**
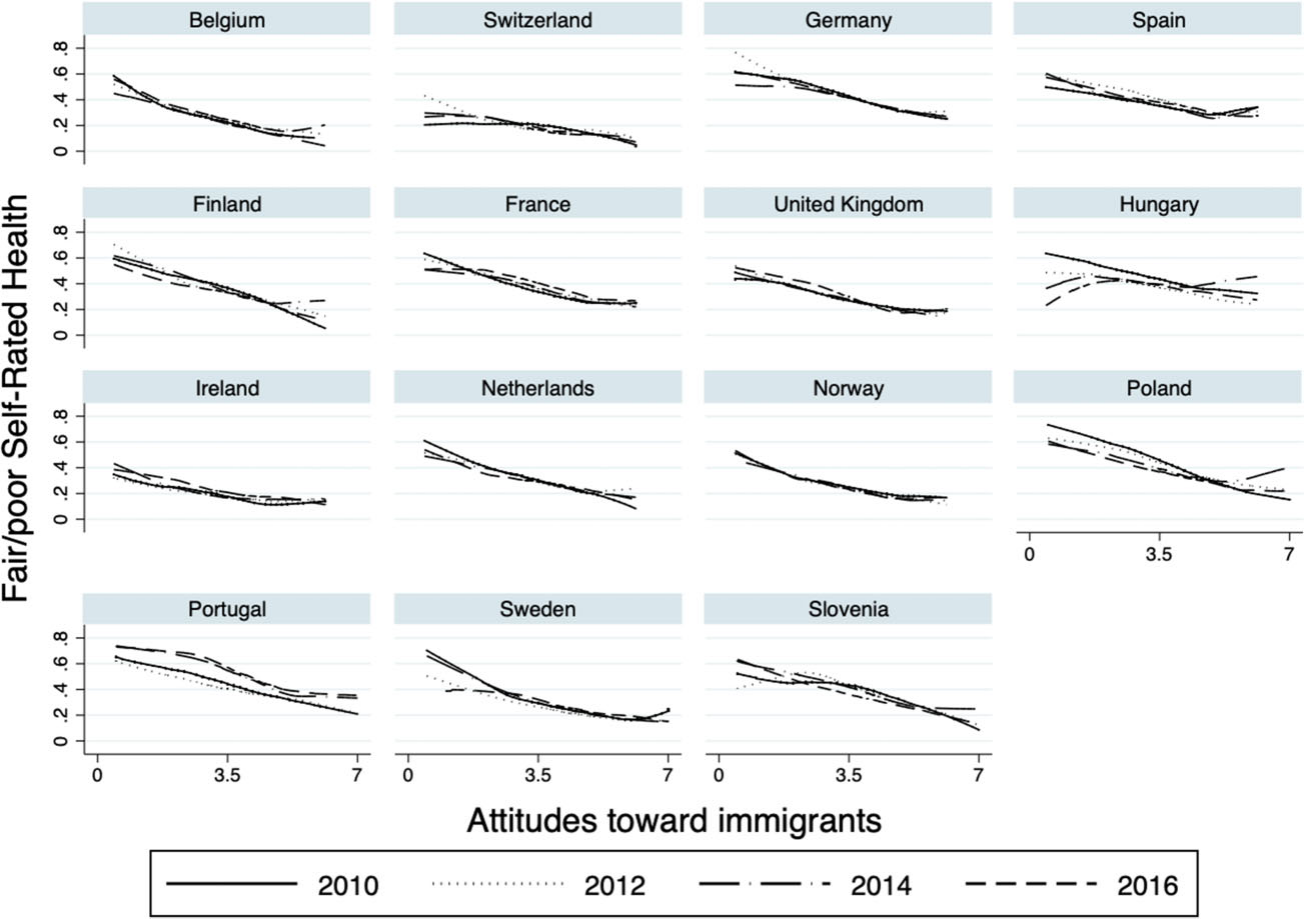
Relationship between fair/poor SRH and attitudes toward immigrants per country and year

However, when assessing this relation by country (figure 2), we find that in some countries the general linear shape does not hold during some years of the survey. For instance, ever since 2014 in Hungary, poor attitudes toward immigrants were related to ‘lower’ odds of reporting fair/poor health, whereas in the same year, better attitudes toward immigrants were detrimental to health. The same happened in 2014 in Poland, in 2010 in Sweden and in 2012 in Slovenia; however, in the latter country in 2014, positive attitudes toward immigrants was not associated with lower odds of reporting fair/poor health. In Portugal, in the last two analyzed years (i.e. 2014 and 2016) holding negative attitudes toward immigrants was associated with higher odds of reporting fair/poor health, whereas having positive attitudes was not as much as protective for Portuguese individuals’ health as in previous years. For the remaining countries, the general linear shape was maintained across survey years.

We also analyzed the socioeconomic status (SES) of individuals splitting the sample by those hold unfavorable and favorable attitudes toward immigrants by year, as we used repeated cross-sections and different people are being captured at each round of the survey.

Individuals with positive attitudes toward immigrants came from higher SES backgrounds (i.e. higher educated, higher income or better labor situation) across all waves compared to individuals with negative attitudes toward immigrants. However, since 2010, the group expressing negative attitudes has been increasingly drawn from higher SES backgrounds (table 3).

**Table 3:** SES composition of individuals with favorable and unfavorable attitudes toward immigrants by year

European average	2010		2012		2014		2016	
	*P*	*N*	*P*	*N*	*P*	*N*	*P*	*N*
Educational attainment	*N = *15 067	*N = *10 826	*N = *16 777	*N = *10 665	*N = *15 656	*N = *9809	*N = *16 405	*N = *8816
ISCED 0	1.65%	3.81%	1.65%	3.23%	1.07%	2.55%	1.19%	1.83%
ISCED 1	8.21%	18.46%	7.36%	18.41%	6.78%	14.85%	6.86%	12.95%
ISCED 2	16.80%	23.00%	16.69%	22.12%	15.55%	21.90%	14.11%	21.57%
ISCED 3	31.85%	35.15%	31.90%	36.41%	30.14%	37.64%	29.63%	40.00%
ISCED 4	5.10%	4.30%	5.68%	4.56%	6.09%	5.09%	5.98%	5.01%
ISCED 5	8.85%	5.71%	9.11%	5.89%	9.88%	6.76%	9.43%	6.13%
ISCED 6	12.56%	5.53%	12.18%	5.34%	13.53%	6.10%	14.82%	7.30%
ISCED 7	13.77%	3.79%	14.08%	3.78%	15.60%	4.73%	16.30%	4.82%
ISCED 8	1.22%	0.25%	1.35%	0.27%	1.37%	0.39%	1.68%	0.39%
Labor market situation	*N = *15 064	*N = *10 813	*N = *16 741	*N = *10 641	*N = *15 654	*N = *9772	*N = *16 391	*N = *8819
Employee	79.49%	79.02%	79.67%	78.74%	79.70%	80.18%	80.38%	80.12%
Self-employed	10.59%	10.73%	10.98%	10.67%	11.38%	10.63%	11.39%	11.38%
Family business	1.27%	1.40%	1.71%	1.94%	1.60%	1.83%	2.02%	1.60%
Other situation	8.64%	8.86%	7.64%	8.66%	7.31%	7.36%	6.21%	6.89%
Perceptions about income	*N = *15 001	*N = *10 778	*N* = 16 690	*N = *10 619	*N = *15 595	*N = *9747	*N = *16 356	*N = *8778
Living comfortably on present income	39.08%	23.67%	37.58%	21.88%	42.70%	26.15%	46.12%	27.81%
Coping on present income	44.83%	48.99%	45.29%	46.11%	43.68%	47.90%	42.59%	50.58%
Difficult on present income	12.54%	19.37%	13.51%	22.65%	11.04%	19.46%	9.00%	16.60%
Very difficult on present income	3.55%	7.97%	3.62%	9.37%	2.58%	6.48%	2.29%	5.01%
Main source of household income	*N = *14 934	*N = *10 745	*N = *16 624	*N = *10 560	*N = *15 502	*N = *9657	*N = *16 275	*N = *8736
Wages or salaries	64.12%	51.88%	62.39%	50.15%	62.15%	51.62%	62.04%	53.80%
Income from self-employment (excluding farming)	6.16%	4.75%	5.98%	4.51%	6.12%	4.93%	5.97%	5.06%
Income from farming	1.11%	1.44%	1.15%	1.33%	0.98%	1.59%	0.98%	1.76%
Pensions	20.87%	31.74%	22.03%	32.73%	22.70%	32.35%	23.48%	31.02%
Unemployment/redundancy benefit	2.66%	4.69%	3.12%	4.96%	2.31%	3.22%	2.06%	2.92%
Any other social benefits or grants	3.09%	4.11%	3.23%	4.62%	3.45%	4.42%	3.35%	3.80%
Income from investments, savings etc.	0.50%	0.34%	0.69%	0.52%	0.80%	0.60%	0.77%	0.53%
Income from other sources	1.51%	1.05%	1.41%	1.18%	1.49%	1.26%	1.33%	1.11%

Note: Individuals who obtained a score below the arithmetic mean (3.75) of the variable ‘attitudes toward immigrants’ were included in the group of people with ‘negative attitudes’ (*N*), and those who obtained a score above 3.75 were included in the group of people with ‘positive attitudes’ (*P*).

**Figure 3:**
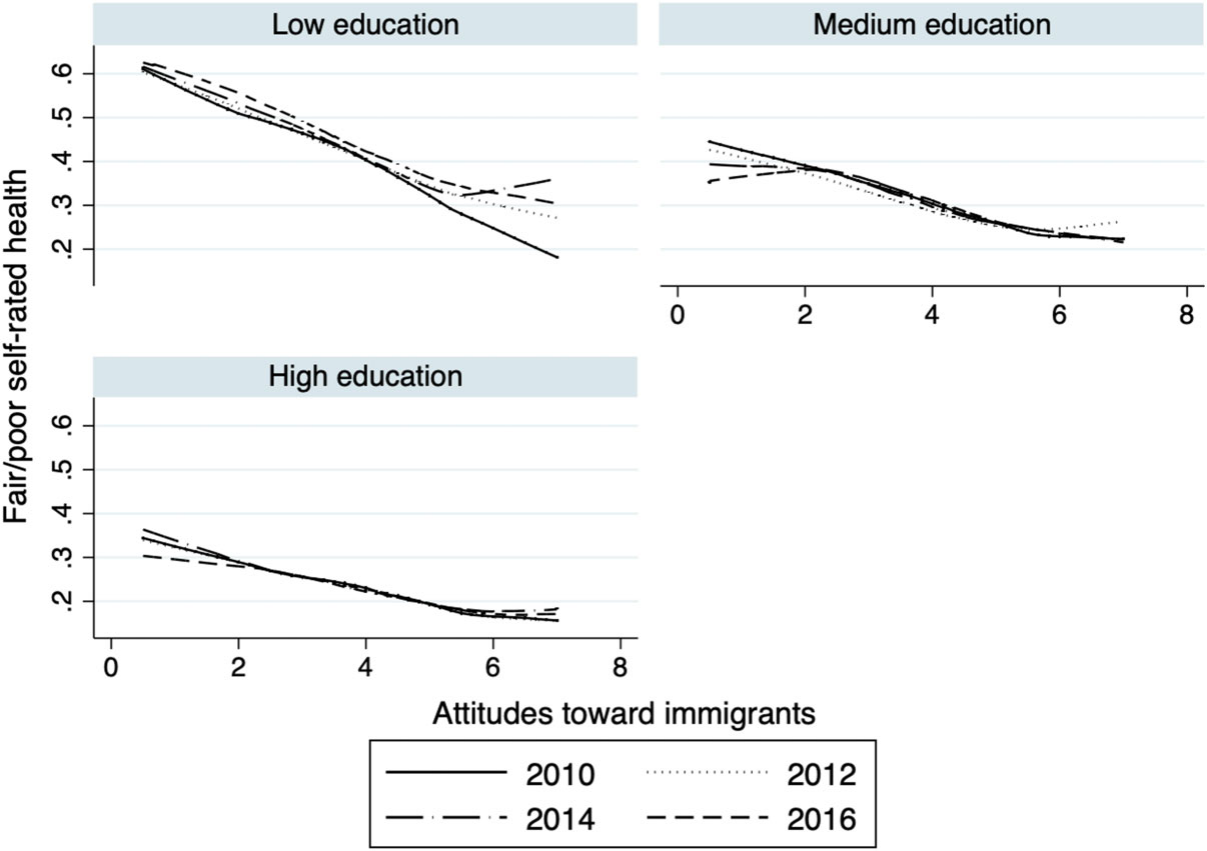
Relationship between fair/poor SRH and attitudes toward immigrants per educational attainment and year Note: Low education includes ISCED 0, 1 and 2 categories; medium education includes ISCED 3 and 4 categories; high education includes ISCED 5, 6, 7 and 8 categories.

We also found that, regardless of individuals’ educational background, individuals with poorer attitudes toward immigrants reported worse SRH (figure 3). However, the slope of the gradient between fair/poor SRH and attitudes toward immigrants was smaller among high education groups. Thus, the trend shown in figure 1, i.e. the slope of the association between attitudes toward immigrants and fair/poor SRH becoming progressively shallower over time, is likely to be due (partly) to a compositional effect whereby more people who report unfavorable attitudes toward immigrants (in 2016) come from higher SES backgrounds.

## Discussion

In general, as attitudes toward immigrants become more favorable, the prevalence of reporting fair/poor health is ‘lower’. However, in the most recent years, the shape of this relationship has slightly changed, which may be due to the ‘composition’ of people who have negative attitudes toward immigrants. We checked that all individuals’ SES backgrounds have improved over cross-sections, regardless of their attitudes toward immigrants. Therefore, as more individuals from advantaged backgrounds begin to harbor negative attitudes toward immigrants, the negative association that poor attitudes toward immigrants have on health might be compensated with the positive association that higher SES characteristics have on individuals’ SRH.

Additionally, the correlation that attitudes toward immigrants have on health seem to vary across time and societal context, indicating that the relationship between attitudes toward immigrant and fair/poor SRH may be contingent on where and when data is collected.

During the years analyzed, right-wing populism has risen in different European countries and their political discourse focused on immigration has increased the salience of the issue on the media, leading people to attach more importance to it.^19^ This fact not only bring to light emotions of fear, anger or hostility already held by some individuals toward immigrants, but also promote ‘politics of insecurity’^20^ leading to violence toward minorities, which negatively affects all individuals’ health.

For instance, in Hungary, Poland and Slovenia, we found that in 2014, individuals who hold positive attitudes toward immigrants presented higher odds of reporting fair/poor SRH compared to other years analyzed. Notably, 2014 was the precise year in which far-right political parties won seats in the European parliament from these countries, and their success was very much linked to their political discourse focused on stoking racial and ethnic divisions.^4–6^

Our results also showed that there are more middle class individuals represented in the negative part of the attitude spectrum in recent years, which appears to reflect the increasing appeal of nationalist ideology to individuals from more advantaged socioeconomic backgrounds.^5^^,^^21^^,^^22^

Although this is a descriptive analysis, the results highlight the importance of analyzing attitudes toward ‘others’ on health, as they may be a marker of individuals’ negative emotions, which are related to health. Additionally, our analysis cannot test whether an individual who changes from better to worse attitudes toward immigrants would see his/her health deteriorate, as we have repeated cross-sections and not panel data.

Individuals may radically change their political affiliation due to the position that a political party has on a specific issue in a given moment^23^^,^^24^; however, attitudes toward that issue are stable and hard to change.^19^ Therefore, although it is proven that, in general, negative attitudes toward immigrants are detrimental to health, public policies aimed to improve these attitudes may backfire.

In this context, the information that individuals receive about immigration should be reliable and accurate to avoid ‘fuel the fire’ among prejudiced individuals. Besides, considering that attitudes toward immigration are developed during young ages,^8^ inclusive education policies promoting ‘cross-group friendships’, i.e. friendships between members of stigmatized and non-stigmatized groups (e.g. immigrants, homosexuals, the disabled, etc.), may be a good approach to improve attitudes toward immigrants and other stigmatized groups across time among the population, as this type of friendships reduce prejudice and anxiety, and increase empathy and trust toward ‘others’.^25^^,^^26^

Inclusionary othering, i.e. strengthening connections to others whilst acknowledging differences, has been shown to counteract the corrosive outcomes associated with exclusionary othering (i.e. stereotyping and marginalization).^9^^,^^10^ Furthermore, exclusionary othering detrimentally affects individuals on both sides. For example, in the healthcare setting, exclusionary othering harms not only patients but also those who are involved in health care provision due to poorer communication and quality of care.^27^

Hence, investing in educational interventions that help the othering process to be inclusive rather than exclusive, i.e. helping people to see ‘differences’ as an opportunity instead of a threat, might not only improve all individuals’ health in general, but also health care provision.

Analyzing how external factors that increase the prejudices and/or stereotypes toward ‘others’ impact on the relationship between attitudes toward immigrants and health, as well as the role of individuals’ SES background, will be an interesting topic for further research and better understand this relationship. To our knowledge, this is the first article, which analyzes the relationship between attitudes toward immigrants and SRH, opening a new field of study in the public health research.

*Conflicts of interest*: None declared.

Key points

- Individuals who harbor hostile attitudes toward immigrants also report poorer self-rated health (SRH).
- Countries with more favorable attitudes about immigrants tend to show lower prevalence of fair/poor SRH; however, this relationship has evolved over time.
- The composition of people who have negative attitudes toward immigrants is changing, with more middle class individuals represented in this part of the attitude spectrum in recent years.

## References

[1] Esses VM , Dovidio JF, Jackson LM, Armstrong TL. The immigration dilemma: the role of perceived group competition, ethnic prejudice, and national identity. J Soc Issues 2001;57:389–412.

[2] Shannon MM. Attitudes Towards Immigrants & Support for Government Spending on Health Care. *Professional Report*. Austin: University of Texas at Austin, 2010.

[3] Kwak J , Wallace M. The impact of the great recession on perceived immigrant threat: a cross-national study of 22 countries. Societies 2018;8:52.

[4] Goldman L , Lim MP, Chen Q, et al Independent relationship of changes in death rates with changes in US Presidential voting. J Gen Intern Med 2019;34:363–71. 30187378 10.1007/s11606-018-4568-6 PMC6420486

[5] Algan Y , Guriev S, Papaioannou E, Passari E. The European trust crisis and the rise of populism. Brookings Pap Econ Act 2017:309–82.

[6] Tyson A , Maniam S. Behind Trump’s Victory: Divisions by Race, Gender and Education. Pew Research Center, 2016. Available at: https://www.pewresearch.org/fact-tank/2016/11/09/behind-trumps-victory-divisions-by-race-gender-education/ (20 April 2021, date last accessed).

[7] Backhaus I , Kino S, La Torre G, Kawachi I. Right-wing populism and self-rated health in Europe: a multilevel analysis. J Epidemiol Community Health 2019;73:1116–21. 31554645 10.1136/jech-2018-211995

[8] Kustov A , Laaker D, Reller C. The stability of immigration attitudes: evidence and implications. J Politics 2021;83(4):1478–94.

[9] Canales MK. Othering: toward an understanding of difference. Adv Nurs Sci 2000;22:16–31. 10.1097/00012272-200006000-00003 10852666

[10] Canales MK. Othering: difference understood? A 10-year analysis and critique of the nursing literature. Adv Nurs Sci 2010;33:15–34. 10.1097/ANS.0b013e3181c9e119 20010068

[11] Stephan WG. Intergroup anxiety: theory, research, and practice. Pers Soc Psychol Rev 2014;18:239–55. 24815215 10.1177/1088868314530518

[12] Stephan WG , Stephan CW. Intergroup anxiety. J Soc Issues 1985;41:157–75.

[13] Page-Gould E , Mendoza-Denton R, Tropp LR. With a little help from my cross-group friend: reducing anxiety in intergroup contexts through cross-group friendship. J Pers Soc Psychol 2008;95:1080–94. 18954195 10.1037/0022-3514.95.5.1080

[14] Salmela M , von Scheve C. Emotional roots of right-wing political populism. Soc Sci Inf 2017;56:567–95.

[15] Staicu M-L , Cuţov M. Anger and health risk behaviors. J Med Life 2010;3:372–5. 21254733 PMC3019061

[16] Haukkala A , Konttinen H, Laatikainen T, et al Hostility, anger control, and anger expression as predictors of cardiovascular disease. Psychosom Med 2010;72:556–62. 20410251 10.1097/PSY.0b013e3181dbab87

[17] Jylhä M. What is self-rated health and why does it predict mortality? Towards a unified conceptual model. Soc Sci Med 2009;69:307–16. 19520474 10.1016/j.socscimed.2009.05.013

[18] Benyamini Y , Leventhal EA, Leventhal H. Gender differences in processing information for making self-assessments of health. Psychosom Med 2000;62:354–64. 10845349 10.1097/00006842-200005000-00009

[19] Mader M , Schoen H. The European refugee crisis, party competition, and voters’ responses in Germany. West Eur Polit 2019;42:67–90.

[20] Béland D. Right-wing populism and the politics of insecurity: how President Trump frames migrants as collective threats. Polit Stud Rev 2020;18:162–77.

[21] Cox D , Lienesch R, Jones RP. Beyond Economics: Fears of Cultural Displacement Pushed the White Working Class to Trump. Washington, DC: Immigration Research Library, 2017.

[22] Koltai J , Varchetta FM, McKee M, Stuckler D. Deaths of despair and Brexit votes: cross-local authority statistical analysis in England and Wales. Am J Public Health 2020;110:401–6. 31855481 10.2105/AJPH.2019.305488 PMC7002930

[23] Meyer M , Schoen H. Avoiding vote loss by changing policy positions. Party Polit 2017;23:424–36.

[24] Bonikowski B. Ethno-nationalist populism and the mobilization of collective resentment. Br J Sociol 2017;68:S181–213. 29114869 10.1111/1468-4446.12325

[25] Pettigrew TF , Tropp LR, Wagner U, Christ O. Recent advances in intergroup contact theory. Int J Intercult Relat 2011;35:271–80.

[26] Christ O , Kauff M. Intergroup contact theory. In: Sassenberg K. and Vliek M.L.W (eds.) Social Psychology in Action. Springer International Publishing, 2019:145–61.

[27] Roberts MLA , Schiavenato M. Othering in the nursing context: a concept analysis. Nurs Open 2017;4:174–81. 28694982 10.1002/nop2.82 PMC5500989

